# Fukushimura Brain Bank (FBB) -Based in a private geriatric hospital-

**DOI:** 10.1186/ar3603

**Published:** 2012-02-09

**Authors:** Hiroyasu Akatsu, Akira Hori, Hidechika Okada, Yoshio Hashizume, Takayuki Yamamoto

**Affiliations:** 1 Choju Medical Institute, Fukushimura Hospital, Japan; 2 Institute of Neuropathology, Fukushimura Hospital, Japan

## 

Fukushimura Brain Bank (FBB) was established under the auspices of Fukushimura Hospital, a legally incorporated medical institution. It is managed completely within the private sector.

"Fukushi" is a Japanese word that means welfare and "mura" is a village. We have several buildings for the aged and disabled, and about 800 elderly people reside within the complex.

The Fukushimura Hospital was established in 1982 and is managed by the Sawarabi MedicalCooperative. It currently has 487 beds. Our patients mainly have dementia and cerebrovascular problems. The hospital plays a pivotal role within the village and acts as the central facility.

FBB was established in 1990. We have a long history of collecting samples, not only from patients but also from residents of our care houses and nursing homes within the Fukushimura complex. This allows us as medical doctors and researchers to obtain clinical information or blood samples, sometimes even before the onset of illness. In our institute, all clinical and pathological dataare held in the office of individual data management.

In collecting FBB samples, we always keep in mind future biochemical and molecular analyses and collaborations. The brains are separated into two hemispheres. One hemisphere is fixed in formalin for neuropathological analysis and the other is precisely subdivided into coronary sections and small blocks which are saved in Eppendorf tubes. After samples are photographed, they are frozen on dry ice (slices) and in liquid nitrogen (tubes). Finally, all material is stored at -80 degrees in 9 refrigerators for later use in research.

Although our bank has gone unrecognized in the past, our farsighted efforts have been gaining considerable attention in recent years in Japan. We now have over 20 collaborators and supply more than 30 research institutes with our samples. In addition, our research institute was approved in 2004 by the Japanese Ministry of Education, Culture, Sports, Science and Technology, as one of the non-governmental institutes which is permitted to apply for governmental grants and we became a member of the Comprehensive Brain Science Network in 2010. FBB at the Choju Medical Institute, Fukushimura Hospitalis a unique facility and one of the most active brain banks in the world.

